# Human Milk Adiponectin and Leptin and Infant Body Composition over the First 12 Months of Lactation

**DOI:** 10.3390/nu10081125

**Published:** 2018-08-20

**Authors:** Zoya Gridneva, Sambavi Kugananthan, Alethea Rea, Ching Tat Lai, Leigh C. Ward, Kevin Murray, Peter E. Hartmann, Donna T. Geddes

**Affiliations:** 1 School of Molecular Sciences, M310, The University of Western Australia, Crawley, Perth, WA 6009, Australia; 21141062@student.uwa.edu.au (S.K.); ching-tat.lai@uwa.edu.au (C.T.L.); peter.hartmann@uwa.edu.au (P.E.H.); donna.geddes@uwa.edu.au (D.T.G.); 2 School of Human Sciences, The University of Western Australia, Crawley, Perth, WA 6009, Australia; 3 Centre for Applied Statistics, The University of Western Australia, Crawley, Perth, WA 6009, Australia; alethea.rea@uwa.edu.au; 4 School of Chemistry and Molecular Biosciences, The University of Queensland, St. Lucia, Brisbane, QLD 4072, Australia; l.ward@uq.edu.au; 5 School of Population and Global Health, The University of Western Australia, Crawley, Perth, WA 6009, Australia; kevin.murray@uwa.edu.au

**Keywords:** adipokines, adiponectin, leptin, breastfeeding, infant, body composition, bioelectrical impedance spectroscopy, ultrasound skinfolds, human milk, lactation

## Abstract

Human milk (HM) adipokines may influence infant feeding patterns, appetite regulation, and body composition (BC). The associations between concentrations/calculated daily intakes (CDI) of HM adipokines in the first 12 months postpartum and maternal/term infant BC, and infant breastfeeding parameters were investigated. BC of breastfeeding dyads (*n* = 20) was measured at 2, 5, 9, and/or 12 months postpartum with ultrasound skinfolds (infants) and bioimpedance spectroscopy (infants/mothers). 24-h milk intake and feeding frequency were measured along with whole milk adiponectin and skim and whole milk leptin (SML and WML) and CDI were calculated. Statistical analysis used linear regression/mixed effects models; results were adjusted for multiple comparisons. Adipokine concentrations did not associate with infant BC. Higher CDI of adiponectin were associated with lower infant fat-free mass (FFM; *p* = 0.005) and FFM index (FFMI; *p* = 0.009) and higher fat mass (FM; *p* < 0.001), FM index (FMI; *p* < 0.001), and %FM (*p* < 0.001). Higher CDI of SML were associated with higher infant FM (*p* < 0.001), FMI (*p* < 0.001), and %FM (*p* = 0.002). At 12 months, higher CDI of WML were associated with larger increases in infant adiposity (2–12 month: FM, *p* = 0.0006; %FM, *p* = 0.0004); higher CDI of SML were associated with a larger decrease in FFMI (5–12 months: *p* = 0.0004). Intakes of HM adipokines differentially influence development of infant BC in the first year of life, which is a critical window of infant programming and may potentially influence risk of later disease via modulation of BC.

## 1. Introduction

A major research focus seeks to elucidate the developmental origins of adiposity and obesity and their health outcomes later in life, since convincing evidence exists of early programming effects on obesity and adiposity [1]. Postnatal feeding choices offer a window of opportunity to prevent obesity. Breastfeeding is implicated in the establishment of infant appetite regulation, feeding patterns, and body composition (BC) and is also associated with reduced risk of developing obesity and a range of other chronic noncommunicable diseases (NCD) [2]. The development of infant BC in early life is known to play an important role in the programming of these health outcomes [3], since deviation from the optimal growth trajectory in early infancy may have a significant effect on adult health later in life. Further, the different growth pattern of breastfed infants compared to those formula-fed has been linked to lower rates of obesity [4,5,6,7]. The reduction in risk may be an outcome of multiple synergetic mechanisms associated with human milk (HM) composition [8,9,10], infant breastfeeding patterns, and behavior [11,12,13,14,15], all of which are highly variable between breastfeeding dyads and may influence infant growth and development of BC.

HM is multifunctional fluid shaped by many thousand years of evolution. As well as essential nutrients, it contains immunological and bioactive components which provide nutrition, protection against infections, developmental factors and, most recently discovered, a host of appetite control factors such as HM adipokines leptin and adiponectin [16]. Higher concentrations of HM adiponectin and leptin have an age-related association with infant weight suggesting an active role in energy homeostasis [17,18,19,20].

Although leptin is the most widely studied of HM appetite hormones, the research in this area is limited and has yet to establish clear relationships between HM leptin and infant BC. However, concentrations of this satiety hormone have been examined predominantly in skim milk where the concentration is significantly lower than in whole milk [21,22] and anthropometric measures or body mass index (BMI) were used, rather than BC measurements [20,23]. BMI is a limited index of adiposity that fails to reflect body shape, fat distribution, and density and may lead to misleading conclusions [23,24]. Therefore, a combination of accurate noninvasive methods to measure infant BC in conjunction with comprehensive HM composition is needed [25]. While leptin has been shown to associate positively with maternal adiposity [22,26], the data on the relationship with infant growth and BC are not conclusive [27], due to heterogeneity in studies designs and few longitudinal studies.

Adiponectin is the appetite hormone present in the highest concentrations in HM and is more than 40-times higher than that of leptin [28]. Amongst its various functions adiponectin regulates lipid and glucose metabolism, stimulates food intake, participates in energy balance, and has anti-inflammatory effects [29,30]. HM adiponectin concentrations are positively associated with maternal serum levels [18,31], and generally maternal serum concentrations of adiponectin are inversely related to maternal body weight and BMI [32,33]. Further, some studies show a positive relationship between HM adiponectin and maternal adiposity [17,28,34], while others show no association [35,36,37,38,39,40]. Adiponectin initially was reported to associate negatively with infant growth and lean body mass accretion in earlier months postpartum [17,31,35,41,42], but recent findings of a few longitudinal studies also report positive associations emerging past 4–6 months of life [18,36,43]. These studies support the notion of differential age-related effects of adiponectin, which modulate growth in early development and promote a growth pattern thought to be responsible for the reduced or increased incidence of adult obesity. This reversal of the initial trend in early life is speculated to be related to the cessation of breastfeeding [18]; high HM adiponectin levels may initially downregulate infant growth, and later promote adipogenesis and adipocyte hypertrophy [44], highlighting the necessity to measure the intake of these adipokines.

It is essential to understand the mechanisms by which breastfeeding and HM may impact infant BC, as this will allow for more targeted interventions that may improve infant outcome and reduce infant and adult overweight and obesity. Thus, the aim of this longitudinal study was to investigate relationships of concentrations and daily intakes of HM adiponectin and leptin with anthropometrics and BC of healthy term breastfed infants and their mothers during first 12 months postpartum. Further, exploration of relationships of infant 24-h milk intake and feeding frequency with HM adipokines was carried out.

## 2. Materials and Methods

### 2.1. Study Participants

Breastfed infants (*n* = 20; 10 males, 10 females) of English-speaking, predominantly Caucasian (18 Caucasian, 2 Asian), mothers of higher social-economic status from a developed country were recruited from the community, primarily from the West Australian branch of the Australian Breastfeeding Association. Inclusion criteria were: healthy singletons, gestational age ≥ 37 weeks, exclusively breastfed [45] at 2 and 5 months, and maternal intention to breastfeed until 12 months. Exclusion criteria were: infant factors that could potentially influence growth and development of BC, maternal smoking, and low milk supply. All mothers provided written informed consent to participate in the study, which was approved by The University of Western Australia Human Research Ethics Committee (RA/1/4253, RA/4/1/2639) and registered with the Australian New Zealand Clinical Trials Registry (ACTRN12616000368437).

### 2.2. Study Session

Measurements were made when the infants were 2 and/or 5, 9, and 12 months of age. Participants visited our laboratory at King Edward Memorial Hospital for Women (Subiaco, Perth, WA, Australia) for up to four monitored breastfeeding sessions between March 2013 and September 2015.

At each study session, the infant was weighed prefeed, and then the mother breastfed her infant. Infant bioelectrical impedance spectroscopy (BIS) measurements were made prefeed, unless impractical, then they were taken postfeed [46]. Ultrasound skinfold (US) and anthropometric measurements were made postfeed. Clothing was removed for the measurements except for a dry diaper and a singlet.

Maternal weight, height, and BIS measurements were recorded. Small (1–2 mL) pre-/postfeed milk samples were collected into 5-mL polypropylene vials (Disposable Products, Adelaide, SA, Australia) from the breast/s that the infant was fed from and samples were frozen at −20 °C for biochemical analysis. Current feeding frequency (FFQ) of the infants was self-reported by mothers.

### 2.3. Anthropometric Measurements

Infants weight was determined before breastfeeding using Medela Electronic Baby Weigh Scales (±2.0 g; Medela Inc., McHenry, IL, USA). Infant crown-heel length was measured once to the nearest 0.1 cm using nonstretch tape and a headpiece and a footpiece, both applied perpendicularly to the hard surface. Infant head circumference was measured with a nonstretch tape to the nearest 0.1 cm.

Maternal weight was measured using Seca electronic scales (±0.1 kg; Seca, Chino, CA, USA). Height was self-reported by participants or measured against a calibrated marked wall (accuracy ± 0.1 cm).

Infant and maternal BMI were calculated as kg/m^2^.

### 2.4. Body Composition with Bioelectrical Impedance Spectroscopy

The methods for measuring maternal and infant BC with the Impedimed SFB7 bioelectrical impedance analyzer (ImpediMed, Brisbane, QLD, Australia) as well as equations for calculations of infant BC parameters have been published previously [14]. The within participant coefficient of variation (CV) for maternal %FM was 0.21% [22]. Within participant CV for infant resistance measurements at 50 kHz (R_50_) was 1.5% [46].

### 2.5. Ultrasound Skinfold Measurements

The method for measuring infant skinfolds using the Aplio XG (Toshiba, Tokyo, Japan) ultrasound machine with a 14–8 MHz transducer (PLT-1204BX) and sterile water-based ultrasonic gel (Parker Laboratories Inc., Fairfield, NJ, USA) as well as equations for calculations of infant BC parameters during this study have been published previously [14,25].

### 2.6. Body Composition Indices

The indices of height-normalized BC were calculated for mothers and infants: FM index (FMI) was calculated as FM/length^2^, and FFM index (FFMI) was calculated as FFM/length^2^; both expressed as kg/m^2^ [47].

### 2.7. 24-h Milk Intake and Feeding Frequency

Infant 24-h milk intake (MI) was measured by mothers using the 24-h milk production (MP) protocol, weighing infants at home with the Medela Electronic Baby Weigh Scales pre- and post each breastfeed during a 24-h period plus one breastfeeding, and recording amounts of HM (g) consumed by the infant (including expressed HM if any) [48]. 24-h MI was determined as previously described with potential underestimation of 3–10% [48] and FFQ (meals per 24-h) was recorded [49]. 24-h MI was measured at 3 time points: between 2 and 5 (4.0 ± 1.3) months, when MI is shown to be stable [49], and within two weeks of 9 (9.4 ± 0.3) and 12 (12.2 ± 0.4) months. Given that measuring 24-h MI is not always practical, particularly at the later stages of lactation, mothers were also asked how frequently the infant fed, and self-reported (SR) the typical time between the meals (e.g., each 2 h) during the week prior to the study session as a proxy measure of FFQ.

### 2.8. Calculated Daily Intakes of Adipokines

24-h MI values from the 24-h MP, and HM adipokine concentrations (averaged pre-/postfeed) from samples taken at the study sessions were used for the calculation of daily intakes (CDI). These CDI were considered representative of a typical daily intake due to absence of significant short-term changes in HM adiponectin and leptin concentrations [22].

### 2.9. Sample Preparation

Prior to further analysis, HM samples were thawed for 2 h at room temperature, mixed on Intelli-Mixer RM-2M (ELMI, Riga, Latvia) at 50 revolutions per min in “UU” mode for 15 s, then, after gentle inversion (3 times), aliquoted into 1.5 mL tubes (Sarstedt, Numbrecht, Germany). Pre- and postfeed samples of whole HM were used for measuring whole milk leptin (WML) and whole milk adiponectin (WMA) concentrations. Milk samples were defatted by centrifugation at room temperature in a Beckman Microfuge 11 (Aberdon Enterprise Inc, Elk Grove Village, IL, USA) at 10,000× *g* for 10 min and removing the fat layer by clipping it off together with the top of the tube [50]. Skim HM was used for measuring skim milk leptin (SML). Standard assays were adapted for and carried out using a JANUS workstation (PerkinElmer, Inc., Waltham, MA, USA) and measured on EnSpire (PerkinElmer, Inc., Waltham, MA, USA).

### 2.10. Leptin

Leptin concentration in whole and skim HM was measured using the R&D Systems Human Leptin ELISA DuoSet kit (R & D system, Minneapolis, MN, USA) with a protocol to measure leptin in skim HM optimized by Cannon et al. [51], and further modified for measurement of leptin in skim and whole HM by Kugananthan et al. [21]. Recovery of leptin was 97.1 ± 9.1% (*n* = 10) with a detection limit of 0.05 ng/mL and an inter-assay CV of <7.2%.

### 2.11. Adiponectin

Adiponectin concentration was measured in whole HM using the Biovendor Human Adiponectin Sandwich ELISA kit (Life Technologies, Asheville, North Carolina, NC, USA). WMA recovery was 96.2 ± 3.2% (*n* = 10) with a detection limit of 1 ng/mL and an inter-assay CV of <2.5%.

### 2.12. Statistical Analyses

Data for this analysis came from the longitudinal study, the details of which, including power calculation, have been described previously [14]. During this longitudinal study participants were measured at 4 time points (2 and/or 5, 9, and 12 months). Descriptive statistics are reported as mean ± standard deviation (SD) and range; model parameters as estimates ± SE (standard errors).

The analyses for systematic differences in concentrations and CDI of adipokines at different months after birth used linear mixed model with age as effect factor and mother as a random factor. Differences between each month were analyzed using general linear hypothesis tests (Tukey’s all pair comparisons).

Relationships between: (a) maternal BC and adipokines’ concentrations/CDI, (b) adipokines’ concentrations/CDI and infant BC, (c) adipokines’ concentrations and breastfeeding parameters (24-h MI/FFQ), and (d) FFQ and CDI of adipokines were analyzed using linear mixed effects models. Each adipokine concentration/CDI or infant BC measure/index was considered separately as the response variable, and each model contained fixed effects of infant age (months), a single predictor (either maternal BC measure/index, adipokine concentration/CDI, or breastfeeding parameters), and an interaction between infant age and predictor, as well as a random intercept per participant. If the interaction is not significant results were reported for the same model fitted without the interaction to assist in understanding the nature of the relationship between the predictor and outcome. As interactions between infant sex and BC measurement methods were nonsignificant (*p* > 0.52) [14], reported associations are for combined male and female data.

Relationships between CDI of adipokines measured between 2 and 5, and at 9 and 12 months after birth and changes (Δ) in infant BC and anthropometric parameters between the time points were analyzed using linear regression models.

Owing to the large number of comparisons, a false discovery rate adjustment [52] was applied to the subgroupings of results to the interaction *p*-value if it was less than 0.05 or to the main effect *p*-value; the adjusted significance levels are reported in Results and Tables and set at the 5% level otherwise. Missing data was dealt with using available case analysis. Statistical analysis was performed in R 3.1.2 [53]. Additional packages were used for linear mixed effects models (nlme, lme4 and car) [54,55,56], intra-class correlations (irr) [57], Tukey’s all pair comparisons (multcomp) [58], and graphics (ggplot2) [59].

## 3. Results

### 3.1. Subjects

Twenty-two two infants were recruited; two infants (one male, one female) were excluded from the study after the 2-month visit (commenced weaning; personal circumstances) and one female infant weaned at 6 months and was therefore excluded from further analysis. The 19 remaining infants were breastfed at 2, 5 and 9 months and 17 infants continued to breastfeed at 12 months. Out of 18 infants measured at 12 months, 16 infants (89%) still continued to breastfeed; one male infant ceased breastfeeding 2 weeks before the 12-month appointment and one female infant stopped at 10 months after birth.

Therefore, overall, 6 infants missed one study session and one infant missed two study sessions. Five of these infants were not recruited until 5 months, one infant did not attend the study session at 9 months, and two did not attend the study session at 12 months. Recruitment of participants at the 5 months point was introduced, as many mothers would not commit to a study that required breastfeeding to 12 months, when approached at 2 months.

Overall 80 measures were expected, however some were missing, specifically: infant weight (*n* = 9); infant BC parameters measured with US 2SF, and maternal age, weight, height, BMI, and BC parameters measured with BIS (*n* = 10); infant head circumference (*n* = 11); infant length, BMI, and BC parameters measured with US 4SF, concentrations of WMA, SML, and WML (*n* = 12); infant BC parameters measured with BIS (*n* = 13); self-reported FFQ (*n* = 20). Missing data also occurred due to difficulties with conducting 24-h MI measurements at later stages of lactation. The following measurements from the 60 expected were missing: FFQ from 24-h MP (*n* = 26), 24-h MI, and CDI of WMA, SML, and WML (*n* = 27). Missing data were spread across the time points (Table 1).

Mean maternal age at the start of the study was 33.3 ± 4.7 (24–44) years, mean height was 167.4 ± 7.4 (150–181) cm and mean parity was 2.3 ± 0.9 (1–4). Infant male/female ratio was 1:1, mean birth weight was 3.486 ± 0.498 (2.660–4.455) kg, and mean gestational age was 39.4 (37.6–43) weeks. Demographic, anthropometric, and breastfeeding characteristics measured at the four study sessions are presented in Table 1. The more detailed determinants of maternal and infant BC as well as description of longitudinal changes in infant and maternal BC and breastfeeding parameters, and the associations between them have been reported previously [14].

### 3.2. Breastfeeding Parameters and Milk Components

HM adipokines concentrations and CDI at 4 time points are detailed in Table 2. Concentration of SML, CDI of WMA, SML and WML, 24-h MI, and both SR and MP FFQ decreased across the lactation (see Table 3 for estimates and significances).

### 3.3. Maternal Body Composition and Adipokines

**Figure 1:**
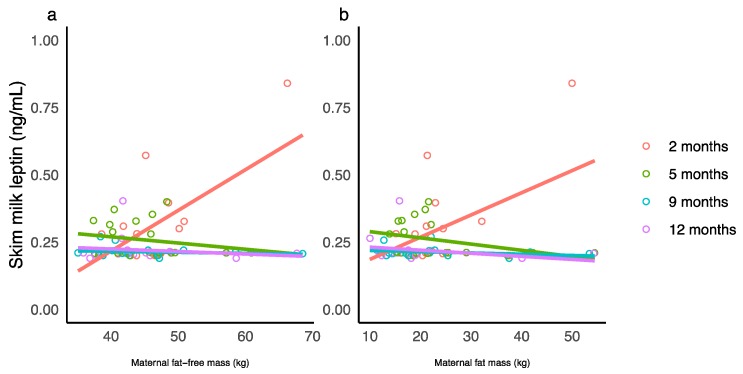
Significant interaction between maternal body composition and the month after birth for skim milk leptin concentration. (**a**) Maternal fat-free mass and (**b**) maternal fat mass measured with bioelectrical impedance spectroscopy. Lines represent linear regression and grouped by the month of lactation.

Significant interactions between maternal characteristics and the month after birth were seen only for SML concentration (Table A1). The changes in slope for maternal characteristics from positive (2 months) to negative (5, 9, and 12) and the decrease in slope indicate that associations between maternal characteristics and SML concentration weaken over the first 12 months of lactation (Figure 1). No significant associations between concentrations of WML and maternal characteristics were seen after adjusting for the false discovery rate. No associations were seen between maternal characteristics and concentrations of WMA as well as CDI of the adipokines (see Table A1 for estimates and significances).

### 3.4. Infant Body Composition and Concentrations of Adipokines

No significant associations between concentrations of adipokines and infant characteristics were seen after adjusting for the false discovery rate (see Table A2 for estimates and significances).

### 3.5. Infant Body Composition and Calculated Daily Intakes of Adipokines

**Figure 2:**
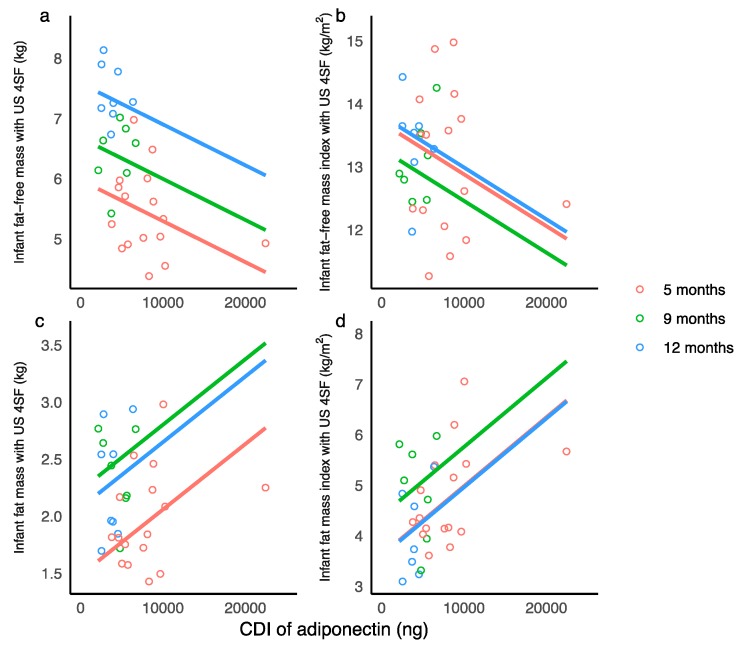
Significant associations between calculated daily intakes (CDI) of whole milk adiponectin. (**a**) Infant fat-free-mass measured with ultrasound 4-skinfolds (US 4SF); (**b**) infant fat-free mass index with US 4SF; (**c**) infant fat mass with US 4SF; (**d**) infant fat mass index with US 4SF. Lines represent linear regression and grouped by the month of lactation.

Higher CDI of WMA were associated with lower infant FFM and FFMI and with an increased infant FM, FMI, and %FM (measured with US 4SF) (Table A3, Figure 2).

**Figure 3:**
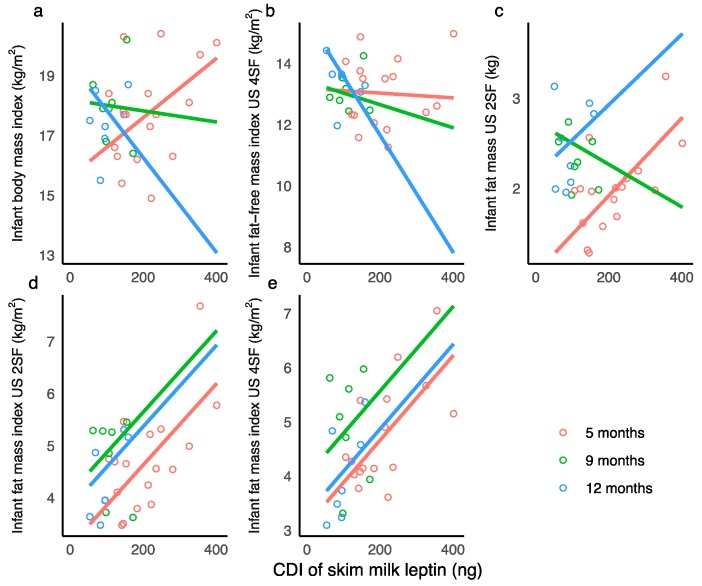
Significant associations between calculated daily intakes (CDI) of skim milk leptin. (**a**) Infant body mass index; (**b**) infant fat-free mass index measured with ultrasound 4-skinfolds (US 4SF); (**c**) infant fat mass with US 2-skinfolds (US 2SF); (**d**) infant fat mass index with US 2SF; (**e**) infant fat mass index with US 4SF. Lines represent linear regression and grouped by the month of lactation.

Higher CDI of SML were associated with an increase in infant BMI at 5 months, a small decrease in at 9 months, and a larger decrease at 12 months. Higher CDI of SML were associated with small decreases in FFMI (US 4SF) at 5 months and 9 months and a larger decrease at 12 months. Higher CDI of SML were associated with an increase in FM (US 2SF) at 5 months, decrease at 9 months, and an increase at 12 months. Higher CDI of SML were associated with an increase in infant FM (US 4SF), FMI (US 2SF, 4SF), and %FM (US 2SF, 4SF) (Figure 3).

No associations were seen between infant characteristics and CDI of WML (see Table A3 for estimates and significances).

### 3.6. Breastfeeding Parameters and Adipokines

A higher concentration of WMA was associated with no change in infant 24-h MP FFQ between 2 and 5 months and a decrease at 9 and 12 months. A higher concentration of WMA was associated with an increase in infant 24-h MI at 5 and 9 months and a decrease at 12 months (Figure S1; Table A4). No significant associations were seen between concentration of WMA and SR FFQ or between concentrations of leptin and breastfeeding parameters. Higher 24-h MP FFQ was associated with an increase in CDI of WML (Figure S2; see Table A4 for estimates and significances). Breastfeeding parameters were not associated with CDI of WMA or SML.

### 3.7. Changes in Infant Characteristics and Calculated Daily Intakes of Adipokines

After accounting for the false discovery rate, significant associations were seen between changes in infant BC (Δ) between the time points and CDI of leptin at the later stages of lactation. Higher CDI of WML at 12 months were associated with larger increase in infant FM (US 2 SF) and %FM (US 2SF) between 2 and 12 months (Table A5), while higher CDI of SML at 12 months were associated with larger decrease in FFMI (US 4SF) between 5 and 12 months (Table A6). No significant associations were seen between infant BC and CDI of WMA after accounting for the false discovery rate (Table A7).

## 4. Discussion

**Figure 4:**
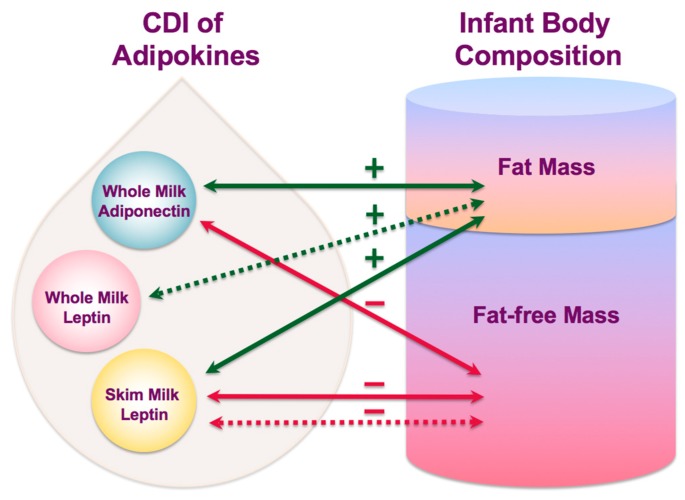
Interconnecting pathways of lactocrine programming of the infant body composition as researched. Solid arrows indicate associations of calculated daily intakes of adipokines with measured body composition parameters and dotted arrows indicate associations with changes in body composition between time points (green—positive associations; red—negative associations). CDI—calculated daily intakes.

This study sheds new light on the complex mechanisms by which breastfeeding may influence infant BC and confer some degree of protection from obesity. For the first time, daily intakes of HM adiponectin and leptin have been associated with development of infant BC and are differentially related to infant FM and FFM (Figure 4) at different stages of lactation. Furthermore, infant FFQ was associated with both the concentration of WMA and CDI of WML emphasizing the critical role of breastfeeding in programming of infant appetite control and growth in the first year of life.

Small bioactive HM peptides believed to be involved in the development of appetite regulation and infant BC include adiponectin and leptin. Whilst several studies have investigated longitudinal changes in both HM leptin and adiponectin, only two studies have measured these hormones at several time points up to 12 months of lactation, one reporting lower concentration of skim milk adiponectin (SMA) [17], and another higher concentrations of SML and WMA [37] at 12 months. We found that although CDI of these adipokines decreased over the 12 months of lactation, the concentrations of both HM leptin and adiponectin measured in whole milk did not change, despite reduction of SML concentration (Section 3.2; Table 3). In this study, higher WML concentrations were associated with higher maternal weight, fat, and lean body mass, but were rendered nonsignificant with adjustment for multiple comparisons (Section 3.3; Table A1). Nevertheless, they are consistent with our earlier whole HM leptin results from a larger cross-sectional cohort [22]. This represents a potential pathway by which HM composition may be improved by maintaining maternal adiposity within the normal range during pregnancy and lactation.

For the first time, we found a higher CDI of adiponectin to be associated with both lower infant lean body mass and higher adiposity (Section 3.5; Table A3). One study has investigated the effect of CDI of adiponectin on infant growth velocity reporting higher CDI of SMA by 3 months in infants with higher weight gain compared with low and normal weight gain groups [10], although no differences in concentrations were detected. We found no association with weight gain in our study, however our study of whole milk is representative of the amount of adiponectin consumed by the infant.

We found a weak relationship between WMA concentration and infant FFM (correction for multiple comparisons eliminated statistical significance) over the first 12 months of lactation (Section 3.4; Table A2). Our results are in contrast to a past study [37], that reported a positive association of WMA with infant weight gain at 6, but not at 12 months. Statistical methods may account for differences where Spearman correlations were employed, with not accounting for stage of lactation or adjusting for multiple comparisons in the previous study. Studies of SMA give conflicting results when compared to WMA studies, with higher skim HM adiponectin concentrations in the first 6 months postpartum being associated with lower weight and lean tissue accretion [36] and lower weight-for-age and weight-for-length z-scores at 6 months of age [17]. Others have shown positive [35,37,39] or no association [38,60,61] with infant growth characteristics. These skim milk studies however showed either positive associations of SMA with weight and adipose tissue accretion at 1 year of life [36] or no associations with weight-for-age and weight-for-length z-scores at 1 year and a positive association during the 2nd year of life [18]. We did not find a positive trend of WMA after 6 months of age calling into question the validity of the skim milk studies.

HM leptin is the most studied adipokine and is implicated in short and long term satiety and regulation of energy intake and body weight [19], still the role of HM leptin in the development of infant BC is yet to be fully understood [27]. For the first time CDI of WML has been linked to infant BC, with higher CDI of both WML and SML associating with greater deposition of adipose tissue (FM, FMI, and %FM), although the results were not comparable (WML, accretion between time points: Section 3.7, Table A5; SML, overall association: Section 3.5, Table A3). These results extend the findings of Kon at al. [10], who reported higher total daily consumption of SML in a group of 3-month-old infants with higher weight gain compared with groups with low and normal weight gain. Other studies report inverse correlations of HM leptin with infant weight or BMI [19,36,42,60,62,63,64], no association [41,65,66], or positive associations [10,35,67].

We found that higher CDI of SML were associated with a lower accrual of infant FFM over 12 months postpartum (Section 3.5; Table A3) and greater reduction in FFMI between 5 and 12 months of age (Section 3.7, Table A6). Leptin from rat adipocytes and osteoblasts is known to both supress and stimulate bone growth [68], and therefore may affect infant lean mass. However, SML concentration/CDI results should be interpreted with caution as associations of SML with infant FFM were different to WML, and SML concentration differed by the month of lactation yet WML concentration did not (Table 3). In addition, skim milk, which excludes the fat and cellular components of HM, has a lower concentration of leptin [21,22,69,70] and is therefore not representative of the milk consumed by the infant. Further study of larger number of infants should be carried out to confirm these new-found relationships.

Breastfeeding frequency and volumes are highly variable between infants and is a reflection of the storage capacity of the breast [49] and likely, of infant appetite regulation [71]. In this study we found a positive relationship between FFQ and CDI of WML (Section 3.7; Table A4), which were also associated with higher FM accretion. Furthermore, the concentration of WMA in our study showed a differential effect on FFQ and 24-h MI (Section 3.7; Table A4), with higher WMA concentrations during earlier months associating with an increase in MI. During later months higher concentrations were associated with reduced FFQ and MI, and may be due to the intake of solids at weaning, although this was not investigated. Recently we have shown increased FFQ was associated with increased 24-h MI, and both of these breastfeeding parameters were related to higher infant adiposity and lower lean body mass [14], suggesting that HM components may differentially influence lean and fat mass compartments, supported by a recent study [72]. Thus, adiponectin and leptin may play active roles in BC development via appetite regulation. These relationships add to the possible pathways of the mechanisms of infant BC regulation.

The strength of this proof-of-concept study is the wide variation of maternal adiposity, that measurements were performed on breastfeeding dyads feeding on demand over 12 months of lactation, and that adipokines were measured in whole HM. The limitations are the small number of 24-h MP at the later stages of lactation, the modest number of participants associated with multiple measurement time points, and the absence of infant dietary data between 6 and 12 months of age. Our population was predominantly Caucasian term healthy fully-breastfed singletons from mothers of higher social-economic status therefore, the results may not be applicable to dyads from other backgrounds.

## 5. Conclusions

These results confirm that the first year of life is a critical window of infant developmental programming and show a differential effect of concentrations and doses of HM leptin and adiponectin on development of infant lean and fat mass during this time. CDI may be a more relevant factor than concentrations when examining the nutritional physiology of the breastfed infant. Given the appetite and BC regulating effects of these adipokines, there is a potential to improve the outcome for the infant through interventions, such as the continuation of breastfeeding during the first year of life and beyond, which may facilitate favorable developmental programming and reduce risk of obesity later in life.

## Figures and Tables

**Table 1:** Participant anthropometric and breastfeeding characteristics.

Characteristic	2 Months ^a^	5 Months ^b^	9 Months ^c^	12 Months ^d^
	Mean ± SD	Mean ± SD	Mean ± SD	Mean ± SD
	(Range)	(Range)	(Range)	(Range)
**Mothers**				
Weight (kg)	78.8 ± 19.3	70.1 ± 17.8	63.0 ± 10.0	64.2 ± 17.3
	(57.5–116.2)	(53.7–115.3)	(50.4–121.9)	(51.4–121.9)
BMI (kg/m^2^)	27.2 ± 5.5	24.8 ± 5.0	22.7 ± 3.9	23.9 ± 5.9
	(20.4–35.5)	(19.0–35.2)	(17.9–37.2)	(18.2–37.2)
**Infants**				
Sex (M/F)	9M/6F	10M/10F	10M/9F	9M/9F
Age (months)	2.04 ± 0.14	5.16 ± 0.22	9.22 ± 0.27	12.26 ± 0.28
	(1.87–2.33)	(4.77–5.47)	(8.83–9.77)	(11.63–12.67)
Length (cm)	58.1 ± 1.9	64.8 ± 2.3	71.7 ± 1.9	73.6 ± 3.2
	(54.2–60.0)	(60.5–69.5)	(66.0–74.0)	(69.0–78.5)
Weight (kg)	5.630 ± 0.660	7.431 ± 1.134	8.836 ± 0.975	9.650 ± 0.618
	(4.420–7.400)	(5.808–9.510)	(6.675–10.095)	(7.165–11.085)
BMI (kg/m^2^)	16.6 ± 1.2	17.6 ± 1.9	17.7 ± 1.7	17.8 ± 0.9
	(14.5–18.1)	(14.9–20.4)	(14.2–20.2)	(13.7–19.2)
Head circumference (cm)	39.7 ± 1.6	42.1 ± 1.5	45.6 ± 1.7	46.6 ± 1.7
	(37.0–42.0)	(40.0–45.9)	(43.0–48.5)	(44.2–49.5)
**Breastfeeding characteristics**				
24-h milk intake (g)	n/a ^e^	818.8 ± 204.9	478.3 ± 154.0	451.1 ± 215.7
		(498–1185)	(300–775)	(255–795)
24-h feeding frequency (MP)	n/a ^e^	8.1 ± 1.4	5.4 ± 1.3	4.4 ± 2.1
		(6–11)	(4–7)	(2–8)
Feeding frequency (SR)	2.3 ± 0.4 ^f^	2.8 ± 0.8	3.7 ± 1.2	5.4 ± 2.9
	(1.5–3.0)	(1.5–4.0)	(2.0–6.0)	(2.2–12.0)

Data are mean ± standard deviation (SD) and ranges. ^a^ *n* = 15; ^b^ *n* = 20; ^c^ *n* = 19; ^d^ *n* = 18. ^e^ Milk intake and feeding frequency as meals per 24-h was determined from 24-h milk production (MP) measured between 2 and 5 months (presented at 5 months here, *n* = 17) and within 2 weeks of 9 (*n* = 6) and 12 months (*n* = 8); n/a—not applicable. ^f^ Maternal self-report (SR) of feeding frequency at the time of the visit as a typical time between meals (e.g., each 2 h) (*n* = 11, *n* = 19, *n* = 17, *n* = 13 at 2, 5, 9, and 12 months respectively). BMI—body mass index.

**Table 2:** Human milk adipokines presented as concentration and 24-h intakes at the months after birth ^a^.

Components	2 Months	5 Months	9 Months	12 Months
	Mean ± SD	Mean ± SD	Mean ± SD	Mean ± SD
	(Range)	(Range)	(Range)	(Range)
**Concentrations ^b^**				
Whole milk adiponectin (ng/mL)	11.14 ± 5.79 ^c^	8.42 ± 1.69	8.44 ± 1.33	11.22 ± 4.22
	(6.61–21.56)	(6.18–22.58)	(6.41–12.86)	(5.66–19.38)
Whole milk leptin (ng/mL)	0.50 ± 0.18	0.49 ± 0.17	0.56 ± 0.11	0.50 ± 0.11
	(0.24–0.77)	(0.23–0.71)	(0.42–0.67)	(0.34–0.74)
Skim milk leptin (ng/mL)	0.34 ± 0.20	0.26 ± 0.08	0.21 ± 0.02	0.21 ± 0.03
	(0.20–0.84)	(0.20–0.40)	(0.19–0.27)	(0.19–0.40)
**CDI ^d^**				
Whole milk adiponectin (ng)	n/a ^e^	7976 ± 4480 ^d^	4446 ± 1645	3922 ± 1431
		(3771–22,439)	(2142–6673)	(2511–6352)
Whole milk leptin (ng)	n/a	362 ± 173	280 ± 73	219 ± 90
		(162–841)	(132–349)	(122–350)
Skim milk leptin (ng)	n/a	200 ± 81	114 ± 38	93 ± 36
		(106–402)	(62–172)	(51–159)

^a^ Milk components’ concentrations and 24-h components’ intakes are presented grouped by the month after birth. ^b^ Concentrations as measured at various months postpartum (*n* = 15, *n* = 20, *n* = 18, and *n* = 15 at 2, 5, 9, and 12 months respectively). ^c^ Data are mean ± SD and ranges. ^d^ CDI of adipokines were calculated between 2 and 5 months (presented at 5 months here, *n* = 17) and within 2 weeks of 9 (*n* = 8) and 12 months (*n* = 8). ^e^ n/a—not applicable.

**Table 3:** Differences by infant age/lactation duration within measured human milk adipokines and breastfeeding parameters ^a^.

Characteristic	Months after Birth						
	5 and 2	9 and 2	12 and 2	9 and 5	12 and 5	12 and 9	*p* Overall
**Milk components**							
Whole milk adiponectin (ng/mL)	−1.01 (0.96) ^b^ 0.72	−1.81 (0.98) 0.25	0.32 (1.05) 0.99	−0.80 (0.90) 0.81	1.33 (0.96) 0.51	2.13 (0.98) 0.13	0.13 ^c^
Whole milk leptin (ng/mL)	−0.04 (0.05) 0.87	0.05 (0.06) 0.74	0.02 (0.05) 0.98	0.08 (0.04) 0.22	0.05 (0.05) 0.65	−0.03 (0.05) 0.92	0.29
Skim milk leptin (ng/mL)	−0.06 (0.03) 0.22	**−0.10 (0.003) ^d^** **0.009**	**−0.10 (0.03)** **0.024**	−0.04 (0.03) 0.52	0.04 (0.03) 0.68	0.01 (0.03) 1.00	**0.012 ^c^**
**Breastfeeding characteristics**							
Feeding frequency (SR) ^e^	0.46 (0.53) 0.82	**1.40 (0.54)** **0.045**	**3.14 (0.58)** **<0.001**	0.94 (0.46) 0.17	**2.69 (0.50)** **<0.001**	**1.75 (0.51)** **0.003**	**<0.001**
Feeding frequency (MP) ^f^	n/a ^g^	n/a ^g^	n/a ^g^	**−2.81 (0.49)** **<0.001**	**−3.71 (0.46)** **<0.001**	−0.90 (0.52) 0.19	**<0.001**
24-h milk intake (g) ^f^	n/a	n/a	n/a	**−325 (64)** **<0.001**	**−376 (64)** **<0.001**	−52 (69) 0.73	**<0.001**
**CDI of milk components**							
Whole milk adiponectin (ng) ^f^	n/a	n/a	n/a	**−3902 (1390)** **0.023**	**−4370 (1390)** **0.010**	−467 (1621) 0.96	**0.004**
Whole milk leptin (ng) ^f^	n/a	n/a	n/a	−100 (58) 0.22	**−147 (58)** **0.044**	−47 (68) 0.77	**0.039**
Skim milk leptin (ng) ^f^	n/a	n/a	n/a	**−103 (29)** **0.004**	**−119 (29)** **<0.001**	−16 (34) 0.89	**<0.001**

^a^ Systematic differences in the measured variables between different months after birth were calculated using general linear hypothesis test (Tukey’s all pair comparisons). ^b^ Data are parameter estimate ± standard error of estimate and *p*-value. ^c^ Overall *p*-value is associated with age as reported in linear mixed model. ^d^ Bold text indicates significant difference (*p* < 0.05) between two time points or overall. ^e^ Feeding frequency was self-reported (SR) by mothers at the time of the visit as an average time between meals (e.g., each 2 h) (*n* = 11, *n* = 19, *n* = 17, and *n* = 13 at 2, 5, 9, and 12 months respectively). ^f^ 24-h milk intake and feeding frequency as meals per 24-h was measured at 24-h milk production (MP) and CDI calculated between 2 and 5 months (presented at 5 months here, *n* = 17) and within 2 weeks of 9 (*n* = 8) and 12 months (*n* = 8). ^g^ Results are not presented for impractical combinations; n/a—not applicable.

## Supplementary Materials

The following are available online at http://www.mdpi.com/2072-6643/10/8/1125/s1, Figure S1: Significant associations between concentration of whole milk adiponectin and (a) infant feeding frequency (meals/24-h); (b) infant 24-h milk intake (g), Figure S2: Significant associations between calculated daily intakes (CDI) of whole milk leptin and infant feeding frequency (meals/24-h) measured during 24-h milk productions.

## Appendix A

**Table A1:** Associations between maternal characteristics and concentrations of human milk adipokines.

Maternal Predictor	2 Months		5 Months		9 Months		12 Months		*p*-Value		
	Intercept (SE)	Slope (SE)	Intercept (SE)	Slope (SE)	Intercept (SE)	Slope (SE)	Intercept (SE)	Slope (SE)	Predictor	Infant Age (Months)	Interaction
**Whole milk leptin (ng/mL)**											
BMI ^d^ (kg/m^2^)	0.35 (0.09) ^a^	0.006 (0.003)	0.33 (0.08)	0.006 (0.003)	0.41 (0.08)	0.006 (0.003)	0.39 (0.08)	0.006 (0.003)	0.045 ^b^	0.17	0.15 ^c^
Weight (kg)	0.35 (0.08)	0.002 (0.001)	0.33 (0.07)	0.002 (0.001)	0.42 (0.07)	0.002 (0.001)	0.39 (0.07)	0.002 (0.001)	0.023	0.18	0.44
FM ^d^ (kg)	0.42 (0.05)	0.003 (0.002)	0.40 (0.05)	0.003 (0.002)	0.49 (0.05)	0.003 (0.002)	0.46 (0.04)	0.003 (0.002)	0.024	0.16	0.45
FFM ^d^ (kg)	0.22 (0.08)	0.002 (0.002)	0.16 (0.08)	0.002 (0.002)	0.11 (0.08)	0.002 (0.002)	0.12 (0.08)	0.002 (0.002)	0.041	0.23	0.46
FMI ^d^ (kg/m^2^)	0.42 (0.06)	0.010 (0.005)	0.39 (0.05)	0.010 (0.005)	0.48 (0.05)	0.010 (0.005)	0.46 (0.05)	0.010 (0.005)	0.038	0.15	0.30
**Skim milk leptin (ng/mL)**											
Weight (kg)	−0.11 (0.11)	0.006 (0.001)	0.35 (0.09)	−0.001 (0.001)	0.23 (0.08)	−0.0002 (0.001)	0.26 (0.08)	−0.001 (0.001)	0.44	0.001	**<0.001**
FM (kg)	0.10 (0.07)	0.008 (0.003)	0.31 (0.05)	−0.002 (0.002)	0.22 (0.05)	−0.001 (0.002)	0.24 (0.05)	−0.001 (0.002)	0.79	0.002	**0.003**
FFM (kg)	−0.39 (0.14)	0.015 (0.003)	0.36 (0.14)	−0.002 (0.003)	0.23 (0.12)	−0.0004 (0.003)	0.26 (0.12)	−0.001 (0.003)	0.14	0.001	**<0.001**

^a^ Parameter estimate ± standard errors (SE); effects of predictors taken from linear mixed effects models that accounted for month after birth and an interaction between month after birth and predictor with a random effect per participant; if the interaction is not significant parameter estimates are taken from a model with no interaction. ^b,c^ Results are presented only for interactions or predictors with raw *p*-values < 0.05; after the false discovery rate adjustment, the interaction/predictor *p*-values were considered to be significant at <0.023 for whole milk leptin (none are significant), at <0.05 for whole milk adiponectin and calculated daily intakes of all adipokines (none are significant, not shown) and skim milk leptin (indicated by the bold text). ^d^ BMI—body mass index; FM—fat mass; FFM—fat-free mass; FMI—fat mass index.

**Table A2:** Associations between concentrations of human milk adipokines and infant characteristics.

Predictor (Concentration, ng/mL)	2 Months		5 Months		9 Months			12 Months			*p*-Value			
	Intercept (SE)	Slope (SE)	Intercept (SE)	Slope (SE)		Intercept (SE)	Slope (SE)		Intercept (SE)	Slope (SE)	Predictor	Infant Age (Months)	Interaction	
**Infant fat-free mass with ultrasound 4 skinfolds (kg)**														
Whole milk adiponectin	4.53 (0.22) ^a^	−0.032 (0.015)	5.76 (0.21)	−0.032 (0.015)		6.77 (0.20)	−0.032 (0.015)		7.58 (0.23)	−0.032 (0.015)	0.025 ^b^	<0.001	0.052 ^c^	
**Infant fat-free mass with bioelectrical impedance spectroscopy (kg)**														
Whole milk leptin	4.53 (0.42)	−0.33 (0.76)	5.49 (0.30)	−0.44 (0.55)		5.01 (0.57)	2.64 (0.55)		5.62 (0.59)	2.77 (1.04)	0.24	<0.001	0.016	
**Infant head circumference (cm)**														
Skim milk leptin	40.30 (0.44)	−1.85 (0.84)	43.30 (0.40)	−1.85 (0.84)		45.80 (0.39)	−1.85 (0.84)		46.80 (0.39)	−1.85 (0.84)	0.028	<0.001	0.37	

^a^ Parameter estimate ± SE; effects of predictors taken from linear mixed effects models that accounted for month after birth and an interaction between month after birth and predictor with a random effect per participant; if the interaction is not significant parameter estimates are taken from a model with no interaction. ^b,c^ Results are presented only for interactions or predictors with raw *p*-values < 0.05; after the false discovery rate adjustment, the interaction/predictor *p*-values were considered to be significant at <0.025 for whole milk adiponectin concentration (none are significant), at <0.016 for whole milk leptin (none are significant) and at <0.028 for skim milk leptin concentrations (none are significant).

**Table A3:** Associations between calculated daily intakes of human milk adipokines and infant characteristics.

Predictor (CDI ^d^, ng)	Between 2 and 5 Months		9 Months		12 Months		*p*-Value		
	Intercept (SE)	Slope (SE)	Intercept (SE)	Slope (SE)	Intercept (SE)	Slope (SE)	Predictor	Infant Age (Months)	Interaction
**Infant head circumference (cm)**									
Whole milk adiponectin	43.00 (0.40) ^a^	−0.00002 (0.00002)	44.60 (0.56)	0.0002 (0.0001)	46.80 (0.59)	−0.0001 (0.0001)	0.27	<0.001	0.026
Infant body mass index (kg/m^2^)									
Whole milk adiponectin	16.90 (0.54)	0.0001 (0.0001)	19.0 (0.96)	−0.0002 (0.0002)	20.10 (1.04)	−0.0006 (0.0002)	0.18 ^b^	0.46	0.016 ^c^
Skim milk leptin	15.60 (0.77)	0.001 (0.003)	18.20 (0.95)	−0.002 (0.008)	19.40 (0.84)	−0.016 (0.007)	0.060	0.21	**0.004**
**Infant fat-free mass with ultrasound 4 skinfolds (kg)**									
Whole milk adiponectin	5.97 (0.24)	−0.0001 (0.00002)	6.68 (0.20)	−0.0001 (0.00002)	7.58 (0.20)	−0.0001 (0.00002)	**0.005**	<0.001	0.80
Infant fat-free mass index with ultrasound 4 skinfolds (kg/m^2^)									
Whole milk adiponectin	13.70 (0.42)	−0.0001 (0.00004)	13.30 (0.36)	−0.0001 (0.00004)	13.80 (0.35)	−0.0001 (0.00004)	**0.009**	0.060	0.056
Skim milk leptin	13.20 (0.59)	−0.0008 (0.003)	13.40 (0.76)	−0.004 (0.006)	15.50 (0.65)	−0.019 (0.006)	0.11	0.10	**0.012**
Whole milk leptin	13.00 (0.47)	0.0002 (0.001)	13.50 (1.08)	−0.002 (0.004)	15.40 (0.79)	−0.008 (0.003)	0.75	0.15	0.036
**Infant fat mass with ultrasound 2 skinfolds (kg)**									
Skim milk leptin	1.06 (0.21)	0.004 (0.001)	2.74 (0.27)	−0.002 (0.002)	2.15 (0.21)	0.004 (0.002)	<0.001	<0.001	**0.007**
**Infant fat mass with ultrasound 4 skinfolds (kg)**									
Whole milk adiponectin	1.48 (0.16)	0.0001 (0.00002)	2.23 (0.14)	0.0001 (0.00002)	2.07 (0.14)	0.0001 (0.00002)	**<0.001**	<0.001	0.19
Skim milk leptin	1.12 (0.21)	0.004 (0.001)	1.99 (0.16)	0.004 (0.001)	1.90 (0.14)	0.004 (0.001)	**<0.001**	<0.001	0.17
**Infant fat mass with bioelectrical impedance spectroscopy (kg)**									
Skim milk leptin	1.57 (0.26)	0.003 (0.001)	1.76 (0.20)	0.003 (0.001)	2.15 (0.19)	0.003 (0.001)	0.025	0.016	0.67
**Infant fat mass with ultrasound 2 skinfolds (%)**									
Skim milk leptin	20.40 (2.13)	0.029 (0.009)	23.80 (1.60)	0.029 (0.009)	22.90 (1.44)	0.029 (0.009)	**0.001**	0.067	0.13
**Infant fat mass with ultrasound 4 skinfolds (%)**									
Whole milk adiponectin	20.80 (1.54)	0.0007 (0.0002)	24.80 (1.30)	0.0007 (0.0002)	21.00 (1.24)	0.0007 (0.0002)	**<0.001**	<0.001	0.12
Skim milk leptin	20.00 (2.17)	0.031 (0.009)	23.80 (1.63)	0.031 (0.009)	20.50 (1.47)	0.031 (0.009)	**0.002**	0.032	0.032>0.43
**Infant fat mass index with ultrasound 2 skinfolds (kg/m^2^)**									
Whole milk adiponectin	4.04 (0.41)	0.0001 (0.00004)	4.63 (0.34)	0.0001 (0.00004)	4.24 (0.34)	0.0001 (0.00004)	0.039	0.20	0.51
Skim milk leptin	3.08 (0.48)	0.008 (0.002)	4.09 (0.35)	0.008 (0.002)	3.82 (0.33)	0.008 (0.002)	**<0.001**	0.002	0.032>0.065
**Infant fat mass index with ultrasound 4 skinfolds (kg/m^2^)**									
Whole milk adiponectin	3.62 (0.38)	0.0001 (0.00004)	4.40 (0.32)	0.0001 (0.00004)	3.60 (0.32)	0.0001 (0.00004)	**<0.001**	0.002	0.10
Skim milk leptin	3.10 (0.50)	0.008 (0.002)	4.01 (0.37)	0.008 (0.002)	3.31 (0.35)	0.008 (0.002)	**<0.001**	0.012	0.29
**Infant fat mass index with bioelectrical impedance spectroscopy (kg/m^2^)**									
Skim milk leptin	3.97 (0.58)	0.005 (0.003)	3.64 (0.43)	0.005 (0.003)	3.88 (0.41)	0.005 (0.003)	0.038	0.71	0.55

^a^ Parameter estimate ± SE; effects of predictors taken from linear mixed effects models that accounted for month after birth and an interaction between month after birth and predictor with a random effect for participant; if the interaction is not significant parameter estimates are taken from a model with no interaction. ^b,c^ Results are presented only for interactions or predictors with raw *p*-values < 0.05; after the false discovery rate adjustment the interaction/predictor *p*-values were considered to be significant at <0.016 for calculated daily intake (CDI) of whole milk adiponectin (indicated by the bold text), at <0.025 for CDI of skim milk leptin (indicated by the bold text) and at <0.036 for CDI of whole milk leptin and (none are significant; data not shown). ^d^ CDI were measured between 2 and 5 months (*n* = 17) and within 2 weeks of 9 (*n* = 8) and 12 months (*n* = 8).

**Table A4:** Associations between human milk adipokines and breastfeeding parameters.

Predictor (Concentration of Adipokine or Breastfeeding Parameter)	Between 2 and 5 Months		9 Months		12 Months		*p*-Value		
	Intercept (SE)	Slope (SE)	Intercept (SE)	Slope (SE)	Intercept (SE)	Slope (SE)	Predictor	Infant Age (Months)	Interaction
**Infant feeding frequency (feeds/24-h) ^d^**									
Whole milk adiponectin (ng/mL)	8.02 (0.82) ^a^	0.010 (0.08)	7.21 (2.36)	−0.23 (0.28)	10.01 (1.90)	−0.63 (0.21)	0.53 ^b^	<0.001	**0.009 ^c^**
**Infant 24-h milk intake (g) ^d^**									
Whole milk adiponectin (ng/mL)	645 (80.40)	17.8 (6.83)	244 (210)	31.5 (24.50)	1120 (161)	−75.3 (16.80)	0.18	<0.001	**<0.001**
**CDI of whole milk leptin (ng) ^d^**									
Feeding frequency (24-h MP) ^f^	1.77 (129.0)	45.0 (15.40)	13.50 (92.60)	45.0 (15.40)	13.60 (80.10)	45.0 (15.40)	**0.004**	0.98	0.74

^a^ Parameter estimate ± SE; effects of predictors taken from linear mixed effects models that accounted for month after birth and an interaction between month after birth and predictor with a random effect per participant; if the interaction is not significant parameter estimates are taken from a model with no interaction. ^b,c^ Results are presented only for interactions or predictors with raw *p*-values < 0.05; after the false discovery rate adjustment, the interaction/predictor *p*-values were considered to be significant at <0.05 for all three adipokines (indicated by the bold text; none are significant for skim milk leptin). ^d^ 24-h milk intake and feeding frequency as meals per 24-h were measured at 24-h milk production (MP) and CDI calculated between 2 and 5 months (*n* = 17) and within 2 weeks of 9 (*n* = 8) and 12 months (*n* = 8).

**Table A5:** Associations between calculated daily intakes of whole human milk leptin at given time points and infant body composition changes between the time points.

Changes in Infant Characteristic	Months after Birth					
	5 and 2	9 and 2	12 and 2	9 and 5	12 and 5	12 and 9
**Calculated daily intake of whole milk leptin (ng) between 2 and 5 months ^e^**						
ΔLength (cm)	0.003 (0.031) ^a^ 0.29 ^b,c^	**0.008 (0.003)** **0.022**	0.003 (0.005) 0.57	−0.0002 (0.002) 0.93	−0.002 (0.003) 0.57	−0.001 (0.003) 0.75
**Calculated daily intake of whole milk leptin (ng) at 9 months ^e^**						
ΔLength (cm)	n/a ^f^	−0.014 (0.022) 0.60	**0.041 (0.004)** **0.007**	−0.005 (0.011) 0.65	0.005 (0.011) 0.67	0.01 (0.012) 0.45
**Calculated daily intake of whole milk leptin (ng) at 12 months ^e^**						
ΔBMI ^d^ (kg/m^2^)	n/a ^f^	n/a ^f^	0.002 (0.01)0.86	n/a ^f^	−0.008 (0.006) 0.22	−0.007 (0.002) 0.019
ΔFat-free mass index US 2SF (kg/m^2^)	n/a	n/a	−0.011 (0.009) 0.34	n/a	**−0.012 (0.004)** **0.040**	−0.005 (0.003) 0.12
ΔFat-free mass index US 4SF (kg/m^2^)	n/a	n/a	−0.011 (0.011) 0.39	n/a	**−0.012 (0.002)** **0.007**	−0.007 (0.004) 0.13
ΔFat mass US 2SF (kg)	n/a	n/a	**0.005 (0.0003)** **0.0006 *****	n/a	0.002 (0.002) 0.22	0.0001 (0.0009) 0.89
ΔFat mass US BIS (kg)	n/a	n/a	**0.005 (0.001)** **0.046**	n/a	−0.001 (0.002) 0.66	−0.0003 (0.002) 0.90
ΔFat mass index US 2SF (kg/m^2^)	n/a	n/a	**0.012 (0.002)** **0.018**	n/a	0.004 (0.004) 0.35	−0.002 (0.002) 0.43
ΔFat mass US 2SF (%)	n/a	n/a	**0.073 (0.004)** **0.0004 *****	n/a	0.024 (0.017) 0.20	−0.002 (0.013) 0.90
ΔFat mass BIS (%)	n/a	n/a	**0.065 (0.015)** **0.049**	n/a	−0.01 (0.021) 0.66	−0.006 (0.028) 0.84

^a^ Parameter estimates ± SE and ^b^ *p*-values for associations between calculated daily intakes (CDI) of whole milk leptin at given time points and the changes (Δ) in measured variables between different months after birth. ^c^ Results are presented only for variables with at least one significant raw *p*-value (*p* <0.05, indicated by the bold text); after the false discovery rate adjustment, the predictor *p*-values were considered to be significant at <0.022 for CDI of whole leptin between 2 and 5 months (none are significant), at <0.007 for CDI at 9 months (none are significant), and at <0.007 for CDI at 12 months (indicated by the bold text and ***). ^d^ BIS—bioimpedance spectroscopy; BMI—body mass index; US 2SF—ultrasound 2-skinfolds; US 4SF—ultrasound 4-skinfolds. ^e^ CDI were measured between 2 and 5 months (*n* = 17) and within 2 weeks of 9 (*n* = 8) and 12 months (*n* = 8). ^f^ Results are not presented for impractical time combinations, n/a—not applicable.

**Table A6:** Associations between calculated daily intakes of skim human milk leptin at given time points and infant body composition changes between the time points.

Changes in Infant Characteristic	Months after Birth					
	5 and 2	9 and 2	12 and 2	9 and 5	12 and 5	12 and 9
**Calculated daily intake of skim milk leptin (ng) between 2 and 5 months ^e^**						
ΔWeight (kg)	0.002 (0.001) ^a^ 0.097 ^b,c^	**0.005 (0.002)** **0.015**	**0.005 (0.002)** **0.026**	0.001 (0.001) 0.46	0.001 (0.002) 0.75	−0.0001 (0.001) 0.84
ΔBMI ^d^ (kg/m^2^)	**0.006 (0.002)** **0.021**	0.004 (0.004) 0.31	0.002 (0.005) 0.63	−0.003 (0.003) 0.33	−0.005 (0.004) 0.23	−0.002 (0.003) 0.53
ΔFat-free mass US 2SF ^d^ (kg)	−0.001 (0.002) 0.47	**0.003 (0.001)** **0.012**	0.003 (0.002) 0.16	0.002 (0.001) 0.25	0.002 (0.002) 0.31	−0.0001 (0.001) 0.89
ΔFat-free mass US 4SF ^d^ (kg)	0.001 (0.001) 0.67	**0.004 (0.001)** **0.010**	**0.005 (0.002)** **0.018**	0.002 (0.001) 0.090	0.003 (0.002) 0.096	0.001 (0.001) 0.40
ΔFat-free mass BIS ^d^ (kg)	0.001 (0.001) 0.21	0.004 (0.002) 0.055	**0.005 (0.001)** **0.013**	0.002 (0.001) 0.098	0.002 (0.002) 0.22	0.0004 (0.002) 0.81
ΔFat mass US 2SF (kg)	**0.003 (0.001)** **0.029**	0.001 (0.001) 0.30	0.002 (0.002) 0.25	−0.001 (0.001) 0.50	−0.001 (0.001) 0.39	0.0002 (0.001) 0.76
ΔFat mass BIS (kg)	**0.002 (0.001)** **0.044**	0.001 (0.002) 0.71	0.001 (0.001) 0.58	−0.002 (0.001) 0.18	−0.002 (0.001) 0.14	−0.0001 (0.002) 0.96
ΔFat mass index US 2SF (kg/m^2^)	**0.007 (0.003)** **0.047**	0.001 (0.003) 0.76	0.001 (0.004) 0.88	−0.004 (0.003) 0.22	−0.005 (0.003) 0.13	−0.001 (0.002) 0.69
ΔFat mass index US 4SF (kg/m^2^)	0.002 (0.001) 0.18	−0.002 (0.004) 0.61	−0.004 (0.005) 0.47	−0.005 (0.003) 0.13	**−0.007 (0.003)** **0.026**	−0.002 (0.001) 0.23
ΔFat mass US 4SF (%)	0.007 (0.009) 0.48	−0.015 (0.017) 0.39	−0.020 (0.020) 0.36	−0.021 (0.014) 0.15	**−0.027 (0.012)** **0.036**	−0.007 (0.008) 0.38
**Calculated daily intake of skim milk leptin (ng) at 9 months ^e^**						
ΔWeight (kg)	n/a ^f^	**0.026 (0.004)** **0.007**	**0.033 (0.008)** **0.025**	−0.005 (0.006) 0.41	−0.006 (0.006) 0.39	−0.0006 (0.002) 0.81
ΔBMI ^d^ (kg/m^2^)	n/a	0.12 (0.04) 0.11	**0.10 (0.02)** **0.035**	−0.01 (0.02) 0.50	−0.02 (0.01) 0.34	−0.004 (0.01) 0.70
ΔFat mass BIS (kg)	n/a	**0.052 (0.012)** **0.046**	0.016 (0.026) 0.60	−0.005 (0.007) 0.47	−0.008 (0.005) 0.13	−0.003 (0.005) 0.60
**Calculated daily intake of skim milk leptin (ng) at 12 months ^e^**						
ΔFat-free mass index US 2SF (kg/m^2^)	n/a ^f^	n/a ^f^	−0.025 (0.010) 0.13	n/a ^f^	**−0.031 (0.007)** **0.005**	−0.013 (0.006) 0.081
ΔFat-free mass index US 4SF (kg/m^2^)	n/a	n/a	−0.022 (0.017) 0.32	n/a	**−0.025 (0.002)** **0.0004 *****	−0.011 (0.009) 0.27
ΔFat mass US 2SF (kg)	n/a	n/a	**0.010 (0.002)** **0.022**	n/a	0.005 (0.004) 0.23	0.002 (0.002) 0.32
ΔFat mass US 2SF (%)	n/a	n/a	**0.135 (0.041)** **0.046**	n/a	0.061 (0.037) 0.14	0.022 (0.029) 0.49

^a^ Parameter estimates ± SE and ^b^ *p*-values for associations between calculated daily intakes (CDI) of skim milk leptin at given time points and the changes (Δ) in measured variables between different months after birth. ^c^ Results are presented only for variables with at least one significant raw *p*-value (*p* <0.05, indicated by the bold text); after the false discovery rate adjustment, the predictor *p*-values were considered to be significant at <0.010 for CDI of skim leptin between 2 and 5 months (none are significant), at <0.007 for CDI at 9 months (none are significant) and at <0.005 for CDI at 12 months (indicated by the bold text and ***). ^d^ BIS—bioimpedance spectroscopy; BMI—body mass index; US 2SF—ultrasound 2-skinfolds; US 4SF—ultrasound 4-skinfolds. ^e^ CDI were measured between 2 and 5 months (*n* = 17) and within 2 weeks of 9 (*n* = 8) and 12 months (*n* = 8). ^f^ Results are not presented for impractical time combinations, n/a—not applicable.

**Table A7:** Associations between calculated daily intakes of whole human milk adiponectin at given time points and infant body composition changes between the time points.

Changes in Infant Characteristic	Months after Birth					
	5 and 2	9 and 2	12 and 2	9 and 5	12 and 5	12 and 9
**Calculated daily intake of adiponectin (ng) between 2 and 5 months ^e^**						
ΔLength (cm)	−0.0001 (0.0001) ^a^ 0.074 ^b,c^	0.0002 (0.0001) 0.079	0.00004 (0.0001) 0.76	**0.0002 (0.0001)** **0.010**	0.0001 (0.0001) 0.47	−0.0001 (0.0001) 0.39
ΔFat-free mass US 4SF ^d^ (kg)	−0.00002 (0.00002) 0.43	**0.0001 (0.00002)** **0.036**	**0.0001 (0.00003)** **0.043**	**−0.0001 (0.00002)** **0.011**	**0.0001 (0.00003)** **0.018**	0.00001 (0.00002) 0.41
ΔFat-free mass index US 2SF (kg/m^2^)	0.00004 (0.0001) 0.42	0.0001 (0.00004) 0.28	**0.0001 (0.00004)** **0.026**	−0.00002 (0.0001) 0.77	0.00004 (0.0001) 0.54	0.00001 (0.00004) 0.17
ΔFat-free mass index US 4SF (kg/m^2^)	0.00002 (0.0001) 0.67	0.0001 (0.00001) 0.092	**0.0002 (0.00004)** **0.009**	0.0001 (0.00001) 0.27	0.0001 (0.0001) 0.057	0.00001 (0.0001) 0.22
ΔFat-free mass index BIS (kg/m^2^)	0.0001 (0.00003) 0.098	0.0001 (0.0001) 0.23	**0.0001 (0.00004)** **0.029**	0.00002 (0.0001) 0.67	0.00004 (0.0001) 0.54	0.00004 (0.0001) 0.44
ΔFat mass US 4SF (kg)	0.00001 (0.00001) 0.50	−0.00003 (0.00002) 0.22	−0.00004 (0.00003) 0.22	−0.00004 (0.00003) 0.17	**−0.0001 (0.00002)** **0.049**	−0.00001 (0.00001) 0.68
ΔFat mass US 4SF (%)	0.0001 (0.0002) 0.62	**−0.001 (0.0002)** **0.044**	−0.001 (0.0003) 0.099	**−0.001 (0.0003)** **0.044**	**−0.001 (0.0002)** **0.007**	**−0.0001 (0.0002)** **0.047**
ΔFat mass index US 4SF (kg/m^2^)	0.00003 (0.00003) 0.36	−0.0001 (0.0001) 0.10	−0.0001 (0.0001) 0.25	−0.0001 (0.0001) 0.059	**−0.0001 (0.0001)** **0.044**	0.000 (0.00003) 0.99
**Calculated daily intake of adiponectin (ng) at 9 months ^e^**						
ΔHead circumference (cm)	n/a ^f^	0.0004 (0.0004) 0.42	−0.0002 (0.0004) 0.64	0.0001 (0.0001) 0.32	−0.0002 (0.0001) 0.066	**−0.0003 (0.0001)** **0.017**
**Calculated daily intake of adiponectin (ng) at 12 months ^e^**						
ΔFat-free mass index US 4SF (kg/m^2^)	n/a ^f^	n/a ^f^	−0.001 (0.001) 0.39	n/a	**−0.001 (0.0002)** **0.020**	−0.0004 (0.0003) 0.23
ΔFat mass index US 2SF (kg/m^2^)	n/a	n/a	**0.001 (0.0001)** **0.005**	n/a ^f^	0.00002 (0.0003) 0.95	0.00002 (0.0002) 0.90
ΔFat mass US 2SF (kg)	n/a	n/a	**0.001 (0.0001)** **0.012**	n/a	−0.00003 (0.0001) 0.82	0.0001 (0.0001) 0.26
ΔFat mass BIS (kg)	n/a	n/a	**0.001 (0.0001)** **0.018**	n/a	−0.0002 (0.0001) 0.17	−0.0001 (0.0002) 0.59
ΔFat mass US 2SF (%)	n/a	n/a	**0.009 (0.002)** **0.013**	n/a	0.001 (0.001) 0.63	0.001 (0.001) 0.46
ΔFat mass BIS (%)	n/a	n/a	**0.007 (0.001)** **0.011**	n/a	−0.002 (0.001) 0.26	−0.001 (0.002) 0.63

^a^ Parameter estimates ± SE and ^b^ *p*-values for associations between calculated daily intakes (CDI) of adiponectin at given time points and the changes (Δ) in measured variables between different months after birth. ^c^ Results are presented only for variables with at least one significant raw *p*-value (*p* < 0.05, indicated by the bold text); after the false discovery rate adjustment, the predictor *p*-values were considered to be significant at <0.007 for CDI of whole milk adiponectin between 2 and 5 months, at <0.017 for CDI at 9 months and at <0.005 for CDI at 12 months (none are significant). ^d^ BIS—bioimpedance spectroscopy; BMI—body mass index; US 2SF—ultrasound 2-skinfolds; US 4SF—ultrasound 4-skinfolds. ^e^ CDI were measured between 2 and 5 months (*n* = 17) and within 2 weeks of 9 (*n* = 8) and 12 months (*n* = 8). ^f^ Results are not presented for impractical time combinations, n/a—not applicable.
