# Supplementary material for: Human Milk Adiponectin and Leptin and Infant Body Composition over the First 12 Months of Lactation
Source: Nutrients. 2018 Aug 20;10(8):1125. doi: 10.3390/nu10081125 (PMC6115716; doi:10.3390/nu10081125)
Supplement: Supplementary file 1 [file nutrients-10-01125-s001.pdf]

Supplementary Materials: Human Milk Adiponectin and Leptin and Infant Body Composition Over the First 12 Months of Lactation

Zoya Gridneva, Sambavi Kugananthan, Alethea Rea, Ching T. Lai, Leigh C. Ward, Kevin Murray, Peter E. Hartmann and Donna T. Geddes

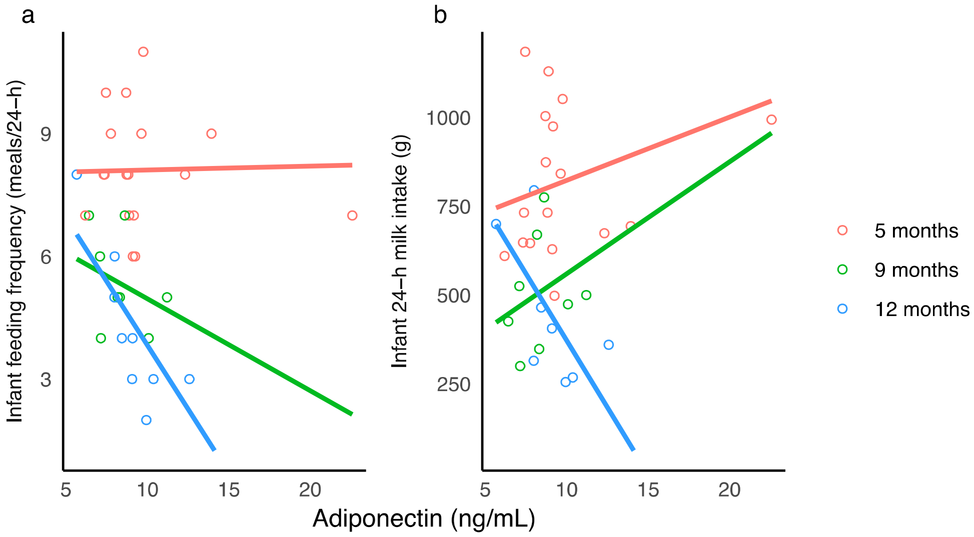

Figure S1. Significant associations between concentration of whole milk adiponectin and (a) infant feeding frequency (meals/24-h); (b) infant 24-h milk intake (g). Lines represent linear regression and grouped by the month of lactation.

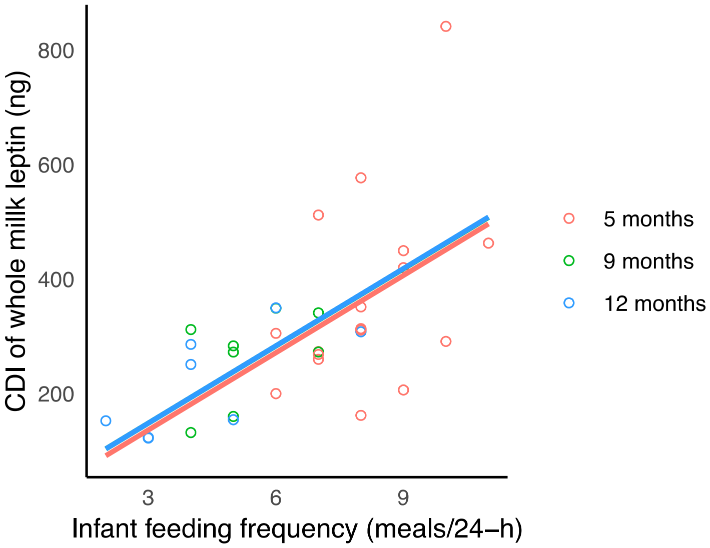

Figure S2. Significant associations between calculated daily intakes (CDI) of whole milk leptin and infant feeding frequency (meals/24-h) measured during 24-h milk productions. Lines represent linear regression and grouped by the month of lactation. The line for 9 months is under the 12 months line due to the similar intercept values.
